# The Conformational Transition Pathways of ATP-Binding Cassette Transporter BtuCD Revealed by Targeted Molecular Dynamics Simulation

**DOI:** 10.1371/journal.pone.0030465

**Published:** 2012-01-17

**Authors:** Jingwei Weng, Kangnian Fan, Wenning Wang

**Affiliations:** Shanghai Key Laboratory of Molecular Catalysis and Innovative Materials, Department of Chemistry and Institute of Biomedical Sciences, Fudan University, Shanghai, People's Republic of China; Russian Academy of Sciences, Institute for Biological Instrumentation, Russian Federation

## Abstract

BtuCD is a member of the ATP-binding cassette transporters in *Escherichia coli* that imports vitamin B_12_ into the cell by utilizing the energy of ATP hydrolysis. Crystal structures of BtuCD and its homologous protein HI1470/1 in various conformational states support the “alternating access” mechanism which proposes the conformational transitions of the substrate translocation pathway at transmembrane domain (TMD) between the outward-facing and inward-facing states. The conformational transition at TMD is assumed to couple with the movement of the cytoplasmic nucleotide-binding domains (NBDs) driven by ATP hydrolysis/binding. In this study, we performed targeted molecular dynamics (MD) simulations to explore the atomic details of the conformational transitions of BtuCD importer. The outward-facing to inward-facing (O→I) transition was found to be initiated by the conformational movement of NBDs. The subsequent reorientation of the substrate translocation pathway at TMD began with the closing of the periplasmic gate, followed by the opening of the cytoplamic gate in the last stage of the conformational transition due to the extensive hydrophobic interactions at this region, consistent with the functional requirement of unidirectional transport of the substrates. The reverse inward-facing to outward-facing (I→O) transition was found to exhibit intrinsic diversity of the conformational transition pathways and significant structural asymmetry, suggesting that the asymmetric crystal structure of BtuCD-F is an intermediate state in this process.

## Introduction

ATP-binding cassette (ABC) transporters constitute a large membrane transport protein family [1], [2]. They utilize the energy of ATP hydrolysis to translocate substrates across the membrane. In organisms, they facilitate nutrient uptake, antigen processing, toxin extrusion [1] and some are also clinically relevant [3], implicated in human genetic diseases such as cystic fibrosis [4], multidrug resistance of cancer cells [5] and atherosclerosis [6]. ABC transporters have at least four domains, including two transmembrane domains (TMDs) and two nucleotide binding domains (NBDs). The cytoplasmic NBDs consist of a RecA-like sub-domain (RSD) and a helical sub-domain (HSD). Two NBDs are arranged head-to-tail, with the RSD of one NBD juxtaposing the HSD of the other NBD, forming two nucleotide binding sites at the interface. By binding and hydrolyzing ATP molecules at the nucleotide binding sites, the NBD dimer switches between the closed and open states [7], [8]. While the sequence and structure of NBDs are highly conserved among different ABC transporters, those of TMDs are highly diverse. One TMD can be composed of 5 or 6 or even more transmembrane (TM) helices. Two TMDs enclose a cavity at their interface which is proposed to be the translocation pathway of substrates. The crystal structures of various ABC transporters show that the substrate translocation pathway at TMD is either at the inward-facing conformation or at the outward-facing conformation [9], [10]. The so-called “alternating-access” mechanism [11] proposed that the translocation pathway switches between the inward-facing and the outward-facing conformations through concerted motions of TMDs and NBDs during the translocation cycle.

**Figure 1:**
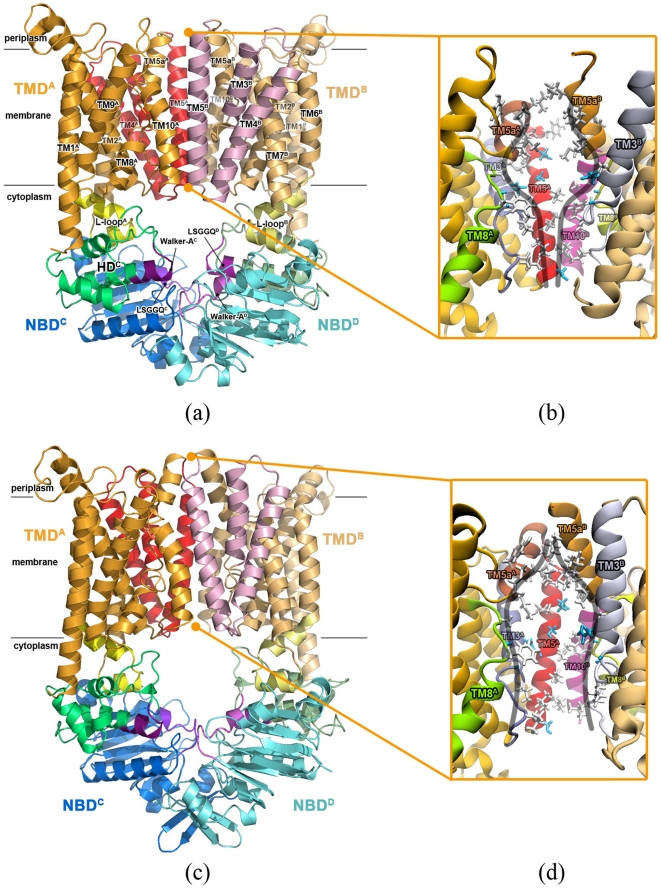
Structures of BtuCD in outward-facing and inward-facing conformations. (a) The ribbon diagram of the crystal structure of BtuCD (PDBID: 1L7V) in an outward-facing conformation, and (b) the close-up view of its translocation pathway, where the hydrophobic residues are shown in stick model. (c) The ribbon diagram of the homology model structure of BtuCD in an inward-facing conformation, and (d) the close-up view of its translocation pathway.

BtuCD is the vitamin B_12_ importer from *Escherichia coli*. It is a dimer of dimer, consisting of two TMDs and NBDs respectively (Figure 1a). Each TMD contains ten TM helices, distinct from the usual five or six TM helices in other ABC transporters. TM5, TM5a, TM8 and TM10 helices and the extended stretches preceding TM3 (exTM3) enclose the translocation pathway at the TMD dimer interface. The side-chains lining the translocation pathway are mainly hydrophobic (Figure 1b). The translocation pathway becomes wider at the middle part, the width of which is large enough to accommodate a vitamin B_12_ molecule [12]. It is referred as the uptake cleft, which accepts the substrate molecules brought by the cognate periplasmic binding protein BtuF [13], [14]. TM6 and TM7 helices of TMD extend into the cytoplasm with the termini folding into two short helices, called L-loops (Figure 1a), which directly interact with NBDs. The NBD dimer of BtuCD adopts a canonical head-to-tail arrangement. Two nucleotide binding sites are formed with the Walker A motifs of one NBD and the signature motifs (LSGGQ motif) of the opposing NBD respectively at the dimer interface.

Different conformations of BtuCD were recently revealed by the high-resolution crystal structures of the intact BtuCD [12] and its homologous protein HI1470/1 [15]. The former represents an outward-facing conformation (outBtuCD thereafter), the translocation pathway of which is occluded at the cytoplasmic side by a hydrophobic constriction formed between TM5 helices and exTM3s. The HI1470/1 importer has very similar fold [15] and biochemical behavior with BtuCD [16]. In the crystal structure of HI1470/1, the translocation pathway is occluded at the periplasmic side and opened at the cytoplasmic side, thereby represents an inward-facing conformation. The conformation of the NBD dimer in HI1470/1 is also different from that in outBtuCD. The HSDs get farther away from each other and the nucleotide binding sites turn into a more open state. These structures strongly support the “alternating access” mechanism, suggesting that the translocation pathway switches between the outward and inward-facing conformations during the translocation cycles. Therefore, revealing the detailed mechanism of this conformational transition is critical for our understanding of the working mechanism of this type of ABC importer.

Besides experimental efforts, molecular dynamics (MD) simulation was also used to study the working mechanism of BtuCD due to its unique strength in exploring protein dynamics [17], [18]. Employing conventional MD simulation method, Oloo *et al* [19] explored the initial stage of the conformational transition of BtuCD. Since the simulation time of dozens of nanoseconds is several orders of magnitude shorter than the time scale of the transportation cycle of about 1 s [20], the whole process of the conformational transition of the transporter can not be monitored [21], [22]. To overcome this limitation, Sonne *et al.* adopted essential dynamics sampling to bias the conformational change along a preset generalized direction, and observed the tilt motion of each TMD subunit, but the conformational changes in the translocation pathway were still limited [23]. To date, the complete reorientation process of the translocation pathway at TMD is still obscure.

In this work, we studied the conformational transition of BtuCD by targeted MD simulation method, in which external force was used to accelerate the transitions between the outward-facing and inward-facing states. Unlike the previous study [23], the external forces in the targeted MD simulation were exerted on both TMD and NBD, ensuring the synchronous conformational transition of the whole protein. In addition, a homology structure of inward-facing BtuCD (inBtuCD thereafter) based on the putative metal-chelate-type transporter HI1470/1, which is more resembled to BtuCD phylogenetically and structurally than the maltose transporter, was used in the simulation. It was found that the outBtuCD-to-inBtuCD transition is initiated by the conformational changes at NBD dimer, and the conformational change of the translocation pathway at TMD begins with the closing of the periplasmic gate. The inBtuCD-to-outBtuCD transition showed significant structural asymmetry, which was not observed in the reverse process.

## Results

### The homology modeling of inBtuCD structure

The inward-facing BtuCD structure (inBtuCD) was obtained by homology modeling based on the crystal structure of HI1470/1 (PDBID: 2NQ2), which shares >30% sequence identity with BtuCD (See Methods for more details). The overall architecture of inBtuCD is very similar with that of the inward-facing HI1470/1 structure (Figure 1c). The C_α_ R.M.S.D. (root-mean-square deviation) between the inBtuCD homology model and the HI1470/1 crystal structure is 0.44 Å. The radius profile of the translocation pathway (Figure S1) illustrates an opening cytoplasmic gate with 4 Å in radius and an occluded periplasmic gate with 0.3 Å in radius. This is a typical inward-facing conformational state, in contrast to the outward-facing state in outBtuCD crystal structure. Despite of the distinct conformations, the inner surface of the translocation channel remains hydrophobic, with both the periplasmic side and the cytoplasmic side lined by hydrophobic side-chains (Figure 1d). The uptake cleft in the middle of the channel is lined by some polar residues and is hardly changed upon the conformational switching of TMD.

With the outBtuCD and inBtuCD structures in hand, we first performed targeted MD simulations to investigate the outBtuCD-to-inBtuCD (O→I) conformational transition, the details of which will be discussed in the following sections.

#### Conformational changes of the NBD dimer is the first step during the O→I transition

Eight 500-ps targeted MD trajectories with different initial velocities were produced for the O→I transition. Since the trajectories share great similarities in the conformational changes, detailed analysis of a representative one was shown below. It should be noted that due to the intrinsic limitation of the targeted MD method, the time scales of the targeted MD trajectories do not equal to the actual time of transition progresses and are not even proportional to. However, previous studies demonstrated that this method can give qualitatively correct pathways of conformational changes [24], [25], [26], [27], [28].

**Figure 2:**
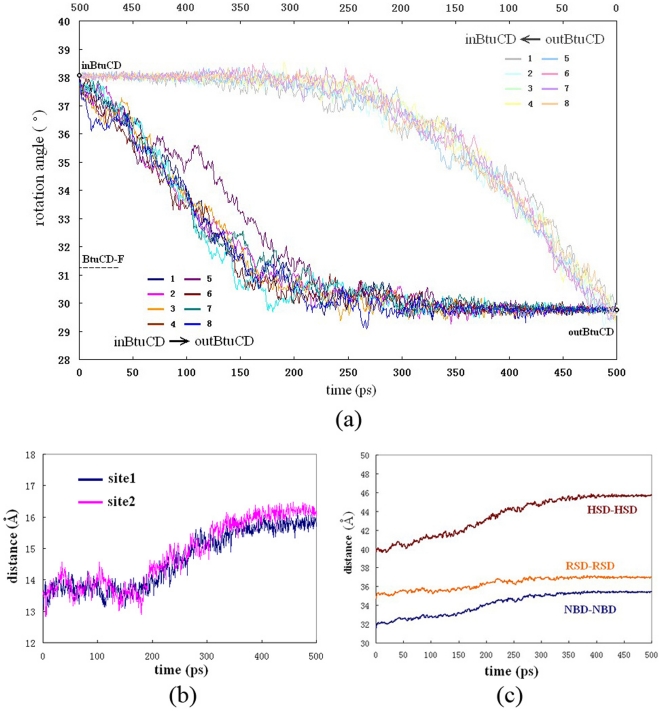
The conformational motions of NBD dimer. (a) The evolution of the spin angle between NBD and TMD during the O→I (light color) and I→O (deep color) transitions. (b) The evolution of the distance between the nucleotide-binding motifs (Walker A and LSGGQ) during O→I transition. (c) The evolution of the distances between the two NBDs and their sub-domains during the O→I transition.

Conformational motions of the NBD dimer can be divided into inter-domain and intra-domain motions. The inter-domain motions are the most evident conformational changes at the beginning of the simulation. The NBD dimer spins relative to TMD dimer along the quasi-C_2_ axis of the whole transporter, changing the relative orientation of the TMD and NBD dimers, as was first revealed by normal mode analysis [29]. The spin angle kept increasing from 30° to 38° in the first 250 ps, after which fluctuated around 38° (Figure 2a). During most of the time of the spin motion, the nucleotide-binding sites at NBD dimer interface remained closed. The distance between the nucleotide-binding motifs (Walker A motif on one NBD and LSGGQ motif on the other) started to increase rapidly from 190 ps and lasted for several hundred picoseconds until the end of the simulation, disrupting the binding sites at the NBD dimer interface (Figure 2b). During this process, there were also obvious intra-domain movements inside each NBD. The HSD subunits underwent rotational movement relative to RSDs, leading to the remarkable increase of the distance between two HSDs after 170 ns (Figure 2c), indicating that the opening of the binding sites is mainly due to the conformational rearrangement of HSD in each NBD domain. In summary, when the simulation begins, the NBD dimer experienced overall motion, spinning around the quasi-C_2_ axis relative to the TMD dimer, followed by intra-domain motions leading to dissociation of the NBD dimer and disruption of the nucleotide-binding sites.

### Reorientation of the translocation pathway began with the closing of the periplasmic side

**Figure 3:**
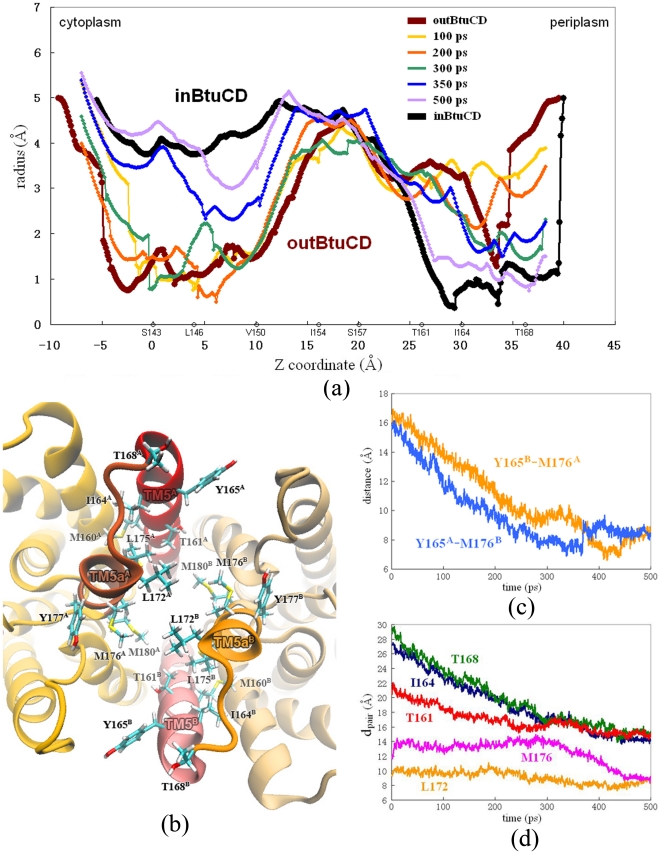
Conformational changes at the periplasmic side of the translocation pathway. (a) Variation of the radius of the translocation pore during the O→I transition. The Z coordinate is along the membrane normal, and the layers denoted with residues are labeled. (b) Close-up top view from the periplasmic side of the translocation pore. Hydrophobic residues are shown with stick model. (c) Evolution of distances between Cα atoms on residues Y165 and M176 during the O→I transition. (d) Evolution of d_pair_s of residues at the perplasmic gate along the simulation trajectory of O→I transition.

To describe the specific positions at the TMD channel, the channel is divided into many layers along the direction of membrane normal, each of which is denoted by a residue within it. For example, T168 layer represents the position at the most periplasmic side of the channel around T168. The conformational changes at TMD happened later than the changes at NBDs and began with the closing of the periplasmic side (Figure 3a). The periplasmic side of the translocation pathway is enclosed by the C-terminus of TM5 helix and the short extension TM5a helix. They form a hydrophobic chamber in the outBtuCD state composed of residues M160, I164 and Y165 on TM5, and L172, L175, M176, Y177, M179 and M180 on TM5a (Figure 3b). When the periplasmic side closes, the helices are drawn together and the residues form extensive hydrophobic interactions around I164 layer. From 100 to 200 ps, the radius of the channel at I164 layer decreased by 1 Å, followed by the contraction at T168 layer (Figure 3a). The distance between TM5^A/B^ and TM5a^B/A^ decreased linearly from the beginning of the simulation and reached the target value at about 300 ps (Figure 3c). At 300 ps, the region between I164 and T168 layers forms a hydrophobic constriction with 1.5 Å in radius and 6 Å in depth (green line in Figure 3a), which occludes the access to the periplasm. During the last 200 ps, the T161 layer contracted evidently and completed the closing of the periplasmic gate (Figure 3a). The final structure at the periplasmic side was almost identical to the target structure (Figure 3a). Upon the closing of the periplasmic gate, the conformational changes of TM5 helices can be viewed as becoming more upright by reducing the tilting angle. This can be monitored by the distances between the two equivalent C_α_ atoms on each TM5 helix (d_pair_). The periplasmic end of TM5 helices experienced largest amplitude of changes, with d_pair_s of I164s and T168s reducing by more than 13 Å (Figure 3d). This was also observed in the previous conventional MD study [19], [22]. The d_pair_s of residues (such as T161) farther from the periplasmic end showed less steep decreases during the first 300 ps of the simulations (Figure 3d).

### The conformational changes at the cytoplasmic side

**Figure 4:**
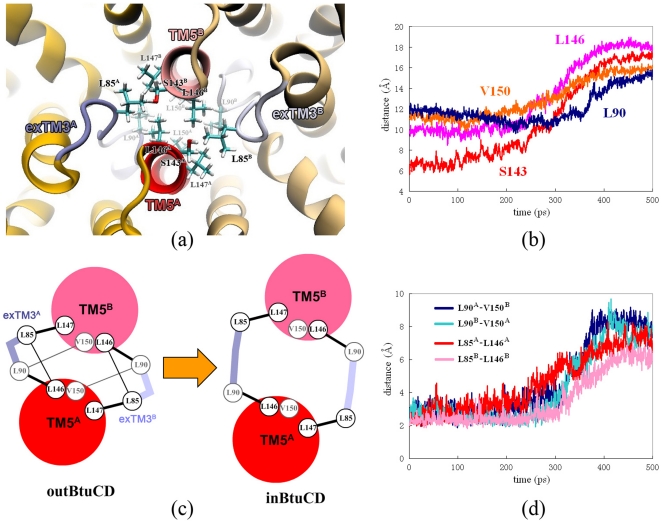
Conformational changes at the cytoplasmic side of the translocation pathway. (a) Close-up bottom view from the cytoplasmic side of the translocation pore. Hydrophobic residues are shown with stick model. (b) Evolution of d_pair_s of residues at the cytoplasmic side along the simulation trajectory of O→I transition. (c) Cartoon representation of the hydrophobic network at the cytoplasmic gate in outBtuCD and inBtuCD. (d) Evolution of minimal distances between residues L90-V150 and L85-L146 during the O→I transition.

The opening of the cytoplasmic side happened later than the closing of the periplasmic side. At 300 ps, the cytoplasmic side was still occluded by S143 and V150 layers with a minimal radius of about 1 Å. There is an intermediate state with both the periplasmic and the cytoplasmic gates occluded around 300 ps (green line in Figure 3a). The cytoplasmic side of the translocation pathway is composed of the N-terminus of TM5 helix and exTM3 segment. In the outBtuCD conformation, they enclose a hydrophobic constriction at the cytoplasmic side which occludes the access of the translocation pathway to the cytoplasm (Figure 4a). The cytoplasmic side began to open after the closing of the periplasmic side, i.e. around 300 ps. The opening of the cytoplasmic side could be largely attributed to the separation of the cytoplasmic ends of TM5 helices. During 300 to 350 ps, the radius at S143 layer increased dramatically. The distance (d_pair_) between the C_α_ atoms of two S143 on TM5 increased by ca 10 Å during the simulation (Figure 4b). The radius at L146 and V150 layers did not change much, keeping the cytoplasmic side occluded. In the last 150 ps, the pore at this region expanded further, but the cytoplasmic side was not fully opened even at the end of the simulation. The pore radius at V150 layer was around 3.0 Å, 1.5 Å less than the targeted value. Therefore, the expanding of the region at V150 layer is the last step and the ‘bottleneck’ for the conformational change of the translocation pathway.

Looking into the details of V150 layer, we found that exTM3 plays an important role in regulating TM5 motions. Two residues on exTM3, L85 and L90, participate in the formation of the hydrophobic core that obstructs the cytoplasmic gate together with L146, L147 and V150 on TM5 helices. Some hydrophobic interactions, such as the side-chain packing between L85^A/B^-L147^B/A^ and L90^A/B^-L146^A/B^ are very stable throughout the simulation, firmly holding the connection between TM5 and exTM3 (Figure 4c). These interactions could prevent two TM5 helices from being too far away from each other or being misarranged during the gate opening. Other hydrophobic interactions, such as L90^A/B^-V150^B/A^ and L85^A/B^-L146^A/B^, however, got disrupted simultaneously at about 300 ps (Figure 4d), loosening the constraints of exTM3 to TM5 helices and facilitating the separation of TM5 helices. The conformation of exTM3 also became more extended, leading to the increase of the distance between L85 and L90 in each segment. The extension of exTM3 stretch and the disruption of the hydrophobic interactions together accelerated the separation of TM5 helices (Figure 4b), resulting in the remarkable expanding of the translocation pore at the cytoplasmic side in the following 50 ps (Figure 3a). TM5 helices and exTM3 stretches constitute a well-designed device at the cytoplasmic side, in which the hydrophobic interactions are elaborately arranged and provide both stability and flexibility. Disruption of the hydrophobic interactions is most likely the rate-limiting step of the outBtuCD-to-inBtuCD transition at the cytoplasmic side.

### The core region in the translocation pathway

**Figure 5:**
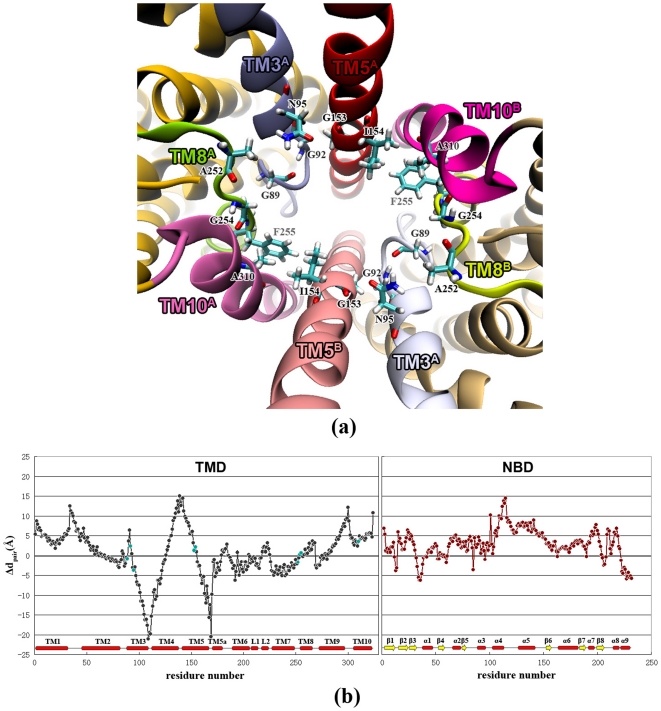
The core region in the middle of the translocation pathway. (a) The residues involved in core region are shown with stick model. TM5a helices are removed for a clear view. (b) Differences of d_pair_s of residues between outBtuCD and inBtuCD states. The residues involved in the core region are colored cyan.

Besides the flexible periplasmic and cytoplasmic ends, there is a rigid part in the middle of the translocation pathway (Figure 3a), to which we refer as the core region. The core region is mainly composed of I154 and S157 layers, spanning from 15 to 30 Å along the membrane normal. It encloses a cavity at the center of TMD with more than 4 Å in radius. The space is large enough to accommodate the substrate molecule, so proposed to be the uptake cleft [12]. The core region consists of G153 and I154 on TM5 helix, G92 and N95 on TM3 helix, G89 on exTM3, A310 on TM10 and A252, G254 and F255 on the extended stretches preceding TM8 (exTM8) (Figure 5a), which are arranged into a ring in the middle of the translocation pathway. The residues in the core region have the smallest d_pair_ differences (Δd_pair_) between inBtuCD and outBtuCD conformations. The Δd_pair_s are always less than 3.7 Å in value and quite small compared with those of the residues in the flexible periplasmic and the cytoplasmic sides along the translocation pathway (Figure 5b). The evolution profiles of d_pair_s in the core region are smooth without abrupt changes (Figure S2), suggesting that the core region is very stable during the conformational transition. Moreover, the core region would also contribute to the stability of TMD dimer interface by holding TM5^A/B^ and TM10^B/A^ helices together. Hydrophobic packing between I154^A/B^, F255^B/A^ and A310^B/A^ closely associates the helices and serves as a hinge point for the tilting motion of TM5 helices in the conformational transition.

### The conformational transition from inward-facing to outward-facing state

We also conducted eight 500 ps targeted MD trajectories for the transition from the inward-facing to the outward-facing conformation (I→O). Unlike the O→I transition, the repeatability of the trajectories in the I→O process is poor. The spatio-temporal order of the conformational changes exhibited diversity among different trajectories. Nevertheless, common features can still be identified among the trajectories. One of the common features is that the spin motion between BtuC and BtuD dimers was always the very first step in the I→O transition, and the eight trajectories gave very similar evolution profiles of the spin angle (Figure 2a). The spin angle began to decrease decently from 38° to about 30° within the first 200 ps and kept constant in the remaining time (Figure 2a). It is interesting that the spin motion between TMD and NBD was also the first step during the O→I transition, implying that the rigid body rotation of TMD and NBD is the prerequisite of the following conformational changes inside TMD and NBD. Comparison of the spin angle variations in the transitions from reverse directions clearly shows that these changes follow different pathways on the potential energy surface (Figure 2a).

The second common feature among the trajectories of the I→O transition is that the translocation pore at TMD always experienced an intermediate state during 300 to 400 ps with both gates occluded just as in the O→I process. The minimal radius of the cavity at either the periplasmic side or the cytoplasmic side was around 2 Å, the size of which is too small for a B_12_ molecule to go through. The BtuCD-F structure supports the idea of a double-occluded cavity by presenting a similar radius profile (Figure S3). The third common feature is that the rigid region at the middle of the translocation pore behaved similarly with the counterpart in the O→I transition, providing structural stability during the large-scale conformational change.

### Structural asymmetry during the conformational transitions

**Figure 6:**
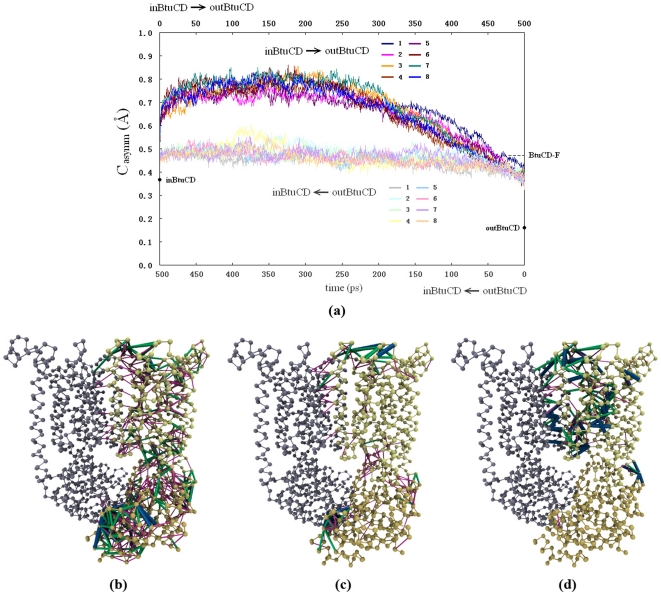
Structural asymmetry in the conformational transitions. (a) Variation of asymmetry coefficient *C_asymm_* along the trajectories of the I→O (deep color) and O→I (light color) transitions. The asymmetry residue pairs (ARPs) in the I→O transition (b), O→I transition (c) and in the BtuCD-F structure (d). ARPs with A_ij_≥2.0 Å are denoted by thick blue lines, those with 1.0≤Aij<2.0 (Å) are denoted by green lines, and those with 0.5≤Aij<1.0 (Å) are denoted by thin pink lines.

During the conformational transitions, the structural symmetry of the homodimer of BtuCD may be broken. In order to quantitatively measure the extent of the conformational asymmetry during O→I or I→O transition, we defined an asymmetry coefficient *C_asymm_* (see Method for the detail), the larger value of which denotes higher degree of structural asymmetry and the value of zero represents strictly symmetric dimer. By this definition, the structures of outBtuCD and inBtuCD are neither exactly symmetric. The *C_asymm_* values of the initial structures of outBtuCD and inBtuCD are 0.16 and 0.37 Å respectively. With respect to the initial static structures, *C_asymm_* increased at the beginning of the simulations, which can be attributed to the thermal motions of the system. During the O→I transition, the *C_asymm_* value remained essentially constant, indicating that the conformational symmetry did not change (Figure 6a). If the structure of BtuCD at the beginning of the trajectory can be considered as essentially symmetric, the evolution of *C_asymm_* value suggests that the O→I transition did not break the structure symmetry of the homodimer. In contrast, the evolution of *C_asymm_* during I→O transition exhibited distinct feature with that of the O→I transition. The protein experiences obvious asymmetric conformational movements in this process despite the fact that the initial and end structures are both symmetric (Figure 6a). All eight trajectories have similar profiles of *C_asymm_* evolution, suggesting that the asymmetry is an intrinsic property of the transition process. This is in agreement with our previous normal mode analysis studies of BtuCD, in which the intrinsic conformational flexibility suggested symmetric conformational change in O→I transition and asymmetric conformational change in I→O transition [29].

In order to explore the spatial distribution of the conformational asymmetry, we defined asymmetrical residue pairs (ARPs) during the conformational transitions by a coefficient *A_ij_* (see Methods). Residue pairs with *A_ij_* more than 0.8 Å were picked out as ARPs and depicted on the structure of BtuCD (Figure 6b,c,d). In the I→O transition, totally 587 ARPs are distributed all over the protein, with 272 ARPs locating at TMD, 263 at NBD and 52 at the TMD-NBD interface (Figure 6b). The regions with high conformation asymmetry include the Walker-A motif in NBD, BtuC-BtuD interface, L-loop, α3-α4 loop, and the periplasmic loops, such as the TM1-TM2 loop and the TM9-TM10 loop. Consistent with the analysis of *C_asymm_*, there are much less ARPs during the O→I transition (totally 179), which are mainly located at the periplasmic side of BtuC subunit, and at the Walker-A motif in BtuD subunit (Figure 6c).

The structural asymmetry in the conformational transitions is reminiscent of the crystal structure of BtuCD-F, in which the TMD domain adopts asymmetric conformation. Analysis of the BtuCD-F structure shows that most of the ARPs locate at TMD region (169 out of 174) and only a few at the BtuC-BtuD interface (Figure 6d). The main characteristics of the conformational asymmetry in BtuCD-F structure resemble those in the conformational transitions manifested in the targeted MD simulations, especially those of the O→I transition. The major difference is that there is no structural asymmetry at NBD in BtuCD-F. Note that the ARP analysis of the targeted MD trajectories reflects the average structural feature during the conformational change, whereas BtuCD-F represents a snapshot in these processes. Another possible explanation is that the difference arises from the effect of BtuF association which has not been considered in the simulation.

## Discussion

Crystal structures and biochemical studies suggest that BtuCD undergoes large scale conformational change during the translocation process, switching between the inward-facing and outward-facing conformations as many other members of ABC transporters. However, BtuCD has several unique characteristics, such as the TMD with 10 TM helices [12], the asymmetry intermediate structure [30], [31], the high basal ATPase activity without the presence of either the cognate periplasmic binding protein or the substrate molecule [20] and the extra high binding affinity with its cognate periplasmic binding protein without the substrate molecule [16]. All these are quite different from other ABC importers, such as the maltose importer MalFGK. Various assumptions of the coupling mechanism of the conformational transitions of BtuCD have been proposed, including the negatively coupling of NBD dimer interface and TM5 cytoplasmic end motion [30], uncoupling between NBDs and TMDs [22], or even an entirely new double-round mechanism [16]. Here we studied the whole cycle of the conformational transitions using targeted MD simulations, revealing several interesting features of the conformational dynamics of BtuCD.

The targeted MD simulations revealed that during the O→I transition of TMD the conformational changes at the periplasmic side are generally ahead of those at the cytoplasmic side, and there exists an intermediate state of TMD with both gates of the translocation pore closed. The priority of the periplasmic gate closure is supported by several lines of experimental and computational studies. First, the crystal structure of BtuCD-F complex presents an occlusion state with its translocation pathway closed to both sides of the membrane [31], consistent with the observations in our simulation as closing the periplasmic gate while holding the cytoplasmic gate occluded would directly lead the protein to the occlusion state. Recently, the conformational dynamics of BtuCD was studied by extensive electron paramagnetic resonance (EPR) experiments with pulsed electron electron double resonance (DEER) technique, as well as continuous wave (cw) EPR spectra [30], [32]. The measured inter-spin distance distributions of residues located at the periplasmic and cytoplasmic ends of TM5 showed that the distance of the periplasmic residue 168 decreased upon docking of BtuF, while those of the cytoplasmic residues would not change until the binding of nucleotide [32]. This suggests that the closed cytoplasmic gate has higher conformational stability than the periplasmic gate. Similar behavior was also reported in the previous conventional MD simulations [22]. In the limited simulation time, partial closing of the periplasmic gate was observed while the ctoplasmic side remained occluded [22]. Therefore, the order of conformational changes of TMD during the O→I transition most likely reflects the intrinsic conformational flexibility of the periplasmic and cytoplasmic gates at the outward-facing state of BtuCD, which is manifested in both experimental and computational studies. In respect of the functional significance, this order of conformational change of TMD ensures that the contracting motion of the periplasmic side would first occlude the access to the periplasm, preventing the substrate molecule in the central cavity from re-diffusing into the periplasm. *In vitro* characterization of substrate binding showed that only free BtuF binds vitamin B_12_ with high affinity, whereas neither BtuCD nor BtuCD-F complex can bind substrate efficiently [16]. The lack of high affinity binding site of substrate on the surface of the translocation pore provides evidence for the functional importance of the conformational change order in the translocation pore. It is interesting to compare with the case of ABC exporter MsbA. Previous targeted MD simulation of the O→I transition of MsbA revealed that unlike BtuCD the conformational rearrangement of the periplasmic side of TMD is the last step in the process [25]. This is also consistent with the requirement of the unidirectional substrate translocation in MsbA because the late conformational change of periplasmic end ensures that the high affinity substrate binding site is only exposed to the cytoplasm when the translocation pore switches to the inward-facing state [33]. It is most likely that the different functional requirements of BtuCD importer and MsbA exporter are partly encoded in the order of conformational changes of TMD during O→I transition. It would be interesting to see whether this order of conformational change is conserved in other types of ABC importers, such as MalFGK and MetNI etc.

The targeted MD simulations also showed that the conformational change at NBD was the first step in the whole process of O→I transition. Similar situation was found in the O→I transition of MsbA exporter [25], suggesting that the nucleotide binding and/or hydrolysis triggers the conformational change at TMD. The EPR experiments showed that in the absence of nucleotide the BtuCD transporter remains in the outward-facing state, which is consistent with the crystal structure of apo-BtuCD [12], [30]. Without nucleotide, the association of BtuF promoted the closing of the periplasmic gate, but the cytoplasmic gate was still remained in an immobile conformation [30] and the inter-spin distances of the cytoplasmic residues did not change [32]. The inter-spin distances of the cytoplasmic residues 141 and 142 increased and the cytoplasmic gate switched into highly mobile conformation only upon addition of nucleotide [30], [32], supporting the idea that the conformational movement at the NBDs is the power stroke of conformational reorientation of TMD. However, the coupling relationship between TMD and NBD in BtuCD is a long debated question. The crystal structures of BtuCD and HI1470/1 demonstrated that the outward-facing state TMD corresponds to a more closed conformation of NBD dimer, and vise versa, whereas the EPR studies showed that the ATP binding led to higher mobility and opening of the cytoplasmic gate at TMD [30], [32]. Uncertainties exist in both structural and EPR experimental studies. Crystal structures were obtained in nucleotide-free states and the effect of crystal packing can not be excluded, while AMPPNP used in the EPR study was previously shown not to be able to mimic ATP to support NBD dimerization [34]. Furthermore, in the EPR experiments S141 and T142 located at the cytoplasmic end of TM5 helices were spin-labeled and used to monitor the cytoplasmic gate movement [32]. Nevertheless, our simulation results demonstrated that the opening of the cytoplasmic gate was limited by the region between L146 and V150 layers, rather than the most cytoplasmic end of the TM5 helices. For example, around 350 ps in the targeted MD trajectory of O→I transition the pore radius in the proximity of S141 and T142 was very close to that in the inward-facing structure, but the cytoplasmic gate still remained closed at L146 layer (Figure 3a). This reminds us that uncertainties exist in choice of the spin probes and it should be more cautious in interpreting the results of EPR experiments. Here, our targeted MD simulation is based on the two crystal structures, which implies the first model of coupling relationship. Under this presumption, the targeted MD trajectories strongly suggest that the separation of NBD dimer initiates the following conformational movement in TMD. The way of signal transduction from NBD to TMD in BtuCD is, however, quite different from that in MsbA. In MsbA, the separation of NBD subunits directly drives the opening of the cytoplasmic gate of TMD [25]. The BtuCD is more like a leverage system, in which L-loop at the TMD-NBD interface serves as a pivot that transfers the separation motion of NBD dimer to the contracting motion at the periplasmic end of TMD. The different mechanical designs may explain why the observed NBD separation in BtuCD is limited while that in MsbA is much larger.

The asymmetric conformation of translocation pore observed in crystal structure of BtuCD-F complex is a unique feature for BtuCD, which has not been found in other ABC transporters. Our previous normal mode analyses of BtuCD and HI1470/1 suggested that symmetric conformational movement is responsible for the O→I transition, whereas the asymmetric conformational movement results in I→O transition. The results of targeted MD simulation are generally consistent with the normal mode analyses. The structural asymmetry in the I→O transition is much more obvious than that in the O→I transition, thereby crystal structure of BtuCD-F may represent a snapshot in the process of I→O transition. However, the asymmetry is confined in the TMD region in the crystal structure of BtuCD-F, while during the I→O transition NBD dimer exhibits significant asymmetric arrangement in addition to the TMDs. The closure of the two nucleotide binding sites did not show any cooperativity in the I→O transition, while in the reverse process the two binding sites were disrupted at almost the same time (Figure 2b). The concomitant asymmetric conformational motions of NBD and TMD were also observed in the normal mode analysis [35]. We note that the targeted MD simulation trajectories of the I→O transition show remarkable diversity. The poor repeatability of the trajectories likely reflects the intrinsic diversity of the conformational transition pathways from inward-facing to outward-facing state, rather than the dependence of the computational protocol which showed good repeatability in the O→I transition. This is reminiscent of the conformational diversity of both periplamic and cytoplasmic ends of TM5 in the absence of BtuF observed in the EPR data analysis [32]. Without BtuF, the transporter BtuCD has a high basal ATPase activity, and experimental kinetic analysis demonstrated that on average BtuCD resides longer in the ADP-bound state than in the ATP-bound or the transition-state-like (Mg^2+^ATP/vanadate) intermediates [16], suggesting that ADP is bound to BtuCD during the restoring process to the outward-facing state.

It is worth noting that the present study did not take into account the periplasmic binding protein BtuF. BtuF was shown to bind with BtuCD with high affinity and dissociate from the transporter upon ATP and substrate binding [16]. However, we do not exactly know whether BtuF is bound to the transporter during the conformational transitions, and there is no structural information of BtuF bound to the outward-facing or inward-facing BtuCD. EPR study demonstrated that BtuF affects the flexibility of periplasmic gate but not the cytoplasmic gate, and normal mode analysis showed that BtuF does not change the main feature of the low frequency normal modes of BtuCD. Revealing the exact role of BtuF on the conformational transitions of BtuCD will await further structural and biochemical studies of the intermediate states in the translocation cycle.

Finally, we should bear in mind, however, that the targeted MD method has its own limitations. Although this method allows us to explore the slow conformational transition (μs to ms) during computational accessible time scale, the relative transition process is not necessarily proportional to actual time. Therefore, it can at most generate qualitatively a correct transition pathway. On the other hand, targeted MD method cannot guarantee that the obtained trajectories follow the globally lowest free energy pathway. Despite these drawbacks, the targeted MD method remains an attractive technique in exploring large scale conformational transition due to its computational efficiency, and many recent applications have shown that this method can provide useful information[24], [25], [26], especially in the close interplay with experimental results.

## Methods

### Conventional molecular dynamics

The simulations were performed with NAMD 2.6 [36] using CHARMM27 force field [37], [38], [39]. TIP3 water model [40] was used for the solvent. Constant temperature was maintained by Langevin dynamics for non-hydrogen atoms with a damping coefficient of 1 ps^−1^. Constant pressure was maintained at 1 bar by the Nosé-Hoover Langevin piston method [41], [42]. The oscillation period was set to 200 fs, the damping time scale was set to 100 fs and anisotropic cell was used. Non-bonded and PME calculations were performed every time step. Short-range interaction was smoothed at 10 Å and truncated at 12 Å. Long-range electrostatic interactions were calculated using the particle mesh Ewald (PME) method [43] with a grid density of 1 Å^−3^. The integration step was set to 2 fs and all bonds involved hydrogen atom was constrained.

The BtuCD protein (PDBID: 1L7V) was inserted into POPE (palmitoyl-oleoyl phosphatidyl-ethanolamine) bilayer by “shrinking” method [44]. The whole system was then solvated and chloride anions were positioned randomly among the solvent to neutralize the system. Periodic boundary conditions were used. There were totally 138423 atoms in a rectangular box with the size of 106×105×118 Å^3^, including 1110 residues, 316 POPE lipids, 20 chloride anions and 27251 water molecules. The whole system was first energy-minimized. Then the solvent was equilibrated for 200 ps with the protein and the lipids fixed. Then the solvent and the lipids were together equilibrated for 3 ns with only the protein constrained. The final structure was used as the initial structure in the targeted molecular dynamics (MD) simulation.

### Homology modeling

The inward-facing BtuCD structure (inBtuCD) was built with MODELLER 9v4 [45], which could automatically derive a set of homology structures after the related structure and its alignment with the target sequence are given to it. The related structure came from the inward-facing crystal structure of HI1470/1 (PDBID: 2NQ2). HI1470/1 shares >30% sequence identity and a very similar topology with BtuCD. The missing fragments in HI1470/1 crystal structure, including the N-terminus of TM1 helix (residue 1 to 4), the extracellular loop 1 (ECL1, residue 20 to 56) and the N-terminus of TM5 helix (residue 140 to 146), were patched up by the corresponding fragments in the BtuCD crystal structure (PDBID: 1L7V). Then MODELLER was employed to produce a homology model assemble. The inBtuCD structure with the lowest DOPE score was picked out for as the target structure in the simulation.

### Targeted molecular dynamics

Targeted MD [46] drives a structure to the target using an external potential which can be described as:
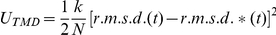
where r.m.s.d.(t) is the instantaneous best-fit r.m.s.d. of the current coordinates to the target coordinates, r.m.s.d.*(t) is the preset r.m.s.d. value for the current time step, and k is the force constant and N the number of targeted atoms. r.m.s.d.*(t) was set to start at 4.11 Å (the best-fit r.m.s.d. between the initial and target structures) and decreased to 0 Å monotonically. The external forces were imposed on all the 1100 C_α_ atoms in BtuCD with a force constant of 5550 kcal/mol/Å^2^. The time step was set to 1 fs, and the coordinates were saved every 500 steps for further analysis.

### Data analysis

The profile of the translocation pathway was calculated using HOLE [47] and the residues enclosing the pathway were also defined by it. The trajectories were analyzed by VMD [48] using tcl scripts. All the 3D graphics were produced by Pymol (http://www.pymol.org) and VMD. The definition of the spin angle between the TMD and NBD dimers [35] utilizes the outBtuCD crystal structure of BtuC and BtuD dimers (referred as oBtuC and oBtuD below) as the reference structures. For a given BtuCD structure, oBtuC and oBtuD dimers are minimally R.M.S.D. overlapped onto the TMD and NBD parts of the structure respectively. As a result, oBtuC and oBtuD dimers are in a new arrangement which reflects the relative positions of TMD and NBD parts in the given structure. The spin angle is defined as the angle between the lines connecting the mass centers of the oBtuC/oBtuD subunits.

The asymmetry coefficient *C_asymm_* as:
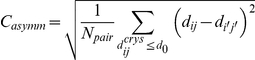
where 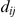 is the distances between the *C_α_* atoms of residue *i* and *j* and 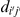 is the counterpart in the opposite half of the protein. N*_pair_* is the number of selected residue pairs.

A residue pair is defined only when its separation distance *d_ij_* is less than the cutoff value *d_0_*, which was set to 8 Å in this study. In BtuCD, 3177 residue pairs are selected, including 1798 residue pairs in BtuC (TMD), 1257 residue pairs in BtuD (NBD), 76 residue pairs across the BtuC-BtuD interface, 30 residue pairs across BtuC-BtuC' interface and 16 residue pairs across BtuD-BtuD' interface.

The coefficient 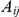 defining ARPs for each residue pair *i-j* during the conformational transition is set as the quadratic mean value of 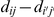 along the trajectory.

## Supporting Information

Figure S1 **The radius profile of the translocation pathway of inBtuCD structure.**
(JPG)

Figure S2 **Variation of d_pair_s of the residues in the core region along the simulation trajectory.**
(JPG)

Figure S3 **The radius profile of the translocation pathway of BtuCDF structure (PDBID: 2QI9).**
(JPG)
