# Supplementary figures and images for: The Conformational Transition Pathways of ATP-Binding Cassette Transporter BtuCD Revealed by Targeted Molecular Dynamics Simulation
Source: PLoS One. 2012 Jan 17;7(1):e30465. doi: 10.1371/journal.pone.0030465 (PMC3260306; doi:10.1371/journal.pone.0030465)

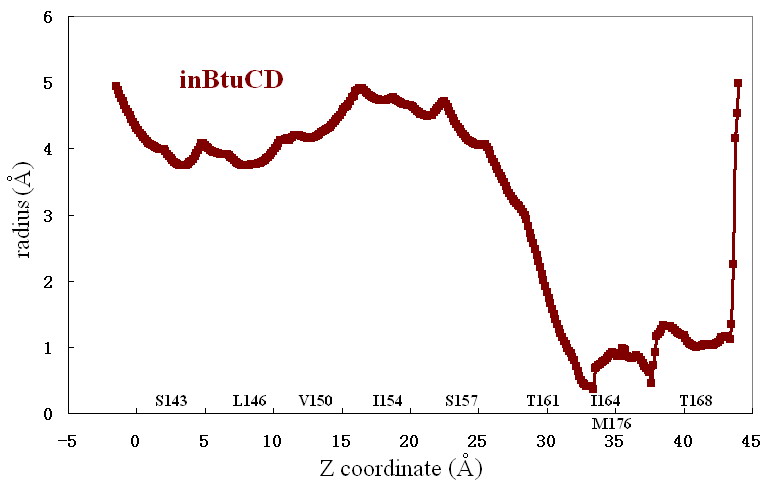

Supplement: Figure S1 — The radius profile of the translocation pathway of inBtuCD structure. (JPG) [file pone.0030465.s001.jpg]

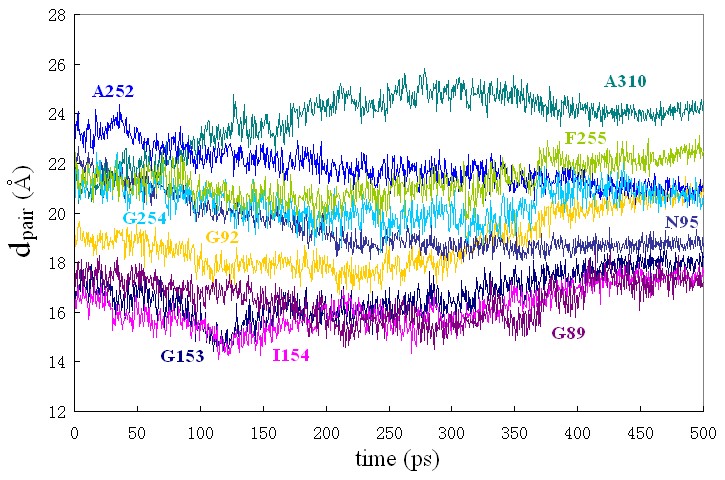

Supplement: Figure S2 — Variation of dpairs of the residues in the core region along the simulation trajectory. (JPG) [file pone.0030465.s002.jpg]

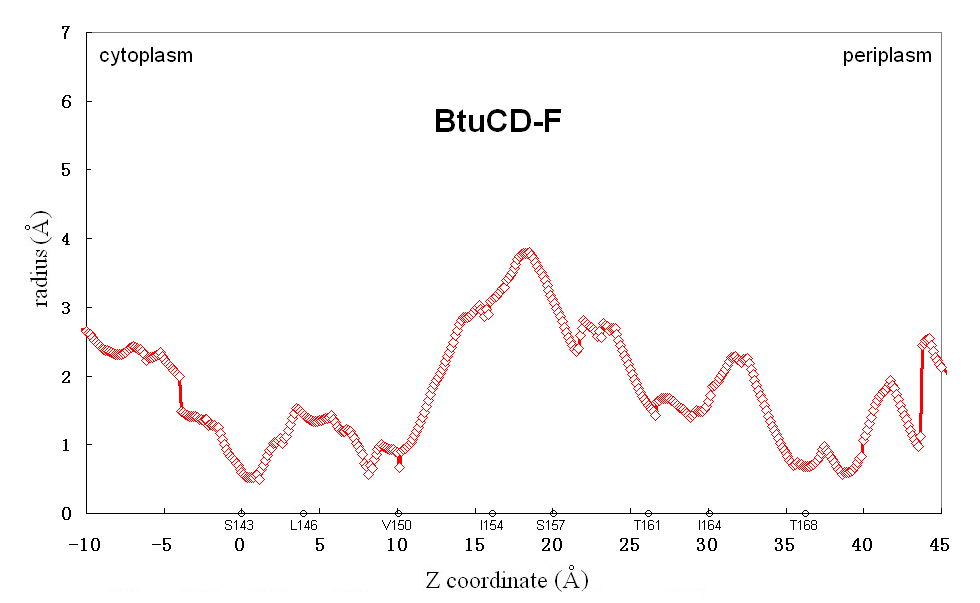

Supplement: Figure S3 — The radius profile of the translocation pathway of BtuCDF structure (PDBID: 2QI9). (JPG) [file pone.0030465.s003.jpg]
